# Prevalence of trypanosomiasis in domesticated animals in Indonesia: A systematic review and meta-analysis

**Published:** 2025-05-25

**Authors:** Lintang Winantya Firdausy, Faisal Fikri, Arya Pradana Wicaksono, Hakan Çalışkan, Muhammad Thohawi Elziyad Purnama

**Affiliations:** 1 Division of Veterinary Medicine, Department of Health and Life Sciences, Faculty of Health, Medicine, and Life Sciences, Universitas Airlangga, Banyuwangi, East Java, 68425, Indonesia; 2 Research Group of Animal Biomedical and Conservation, Faculty of Health, Medicine, and Life Sciences, Universitas Airlangga, Banyuwangi, East Java, 68425, Indonesia; 3 Animal Health Division, Indonesian Horse Veterinarian Association, Surabaya, East Java, 60115, Indonesia; 4 Department of Biology, Faculty of Science, Eskisehir Osmangazi University, Eskisehir, 26040, Turkey; 5 Department of Biology, Graduate School of Natural and Applied Sciences, Eskisehir Osmangazi University, Eskisehir, 26040, Turkey

**Keywords:** domesticated animals, Indonesia, meta-analysis, prevalence, *Trypanosoma evansi*, trypanosomiasis, vector-borne disease

## Abstract

**Background and Aim:**

Trypanosomiasis is a vector-borne parasitic disease with significant implications for animal health and rural livelihoods in Indonesia. Despite surveillance efforts, comprehensive national-level estimates of its prevalence in domesticated animals remain lacking. This study aimed to synthesize the pooled prevalence of trypanosomiasis across Indonesian provinces, identify contributing factors, and assess trends over time using a systematic review and meta-analysis.

**Materials and Methods:**

A systematic search was conducted in seven electronic databases (PubMed, Scopus, Web of Science, ScienceDirect, Cochrane Library, ProQuest, and Google Scholar) for articles published between 1988 and 2024. Eligible studies reported primary prevalence data of trypanosomiasis in domesticated animals within Indonesia. A total of 18 studies with 4,295 samples met the inclusion criteria. Random-effects meta-analysis was performed using R Studio 4.4.2. Subgroup analyses were conducted based on animal host, diagnostic method, province, and study period. Heterogeneity was assessed through I^2^ and τ^2^ statistics, and publication bias was evaluated using Egger’s test and funnel plots.

**Results:**

The pooled prevalence of trypanosomiasis in domesticated animals across Indonesia was 31.23% (95% confidence interval: 24.67–37.78), with considerable heterogeneity (I^2^ = 98.1%). Buffaloes exhibited the highest infection rate at 51.46%, followed by cattle (33.99%), whereas horses and dogs had notably lower rates (<6%). Provinces with the highest reported prevalence included Lampung (75.05%) and Central Kalimantan (75.00%). Enzyme-linked immunosorbent assay was the most frequently used and sensitive diagnostic method. Meta-regression revealed a declining trend over time (p = 0.0002), although high variability persisted between regions and diagnostic tools.

**Conclusion:**

Trypanosomiasis remains endemic among domesticated animals in Indonesia, with a substantial pooled prevalence and marked regional variability. The findings underscore the need for improved surveillance, implementation of standardized diagnostic tools, and integrated vector management strategies. Future research should focus on ecological risk factors, seasonality, and the zoonotic potential of *Trypanosoma evansi* to support evidence-based control interventions.

## INTRODUCTION

Trypanosomes are unicellular hemoflagellate protozoa transmitted by hematophagous arthropods, which infect a wide range of mammalian hosts globally, including humans, particularly in tropical and subtropical regions [1, 2]. Among livestock, trypanosomiasis caused primarily by *Trypanosoma brucei*, *Trypanosoma* *equiperdum*, and *Trypanosoma evansi*, all members of the *Trypanozoon* subgenus poses significant socioeconomic burdens and contribute to substantial reductions in animal productivity [3]. Of these, *T. evansi*, the etiological agent of surra, is the most geographically widespread and demonstrates the greatest host diversity [1]. Since its initial identification in the blood of horses and dromedaries in India in 1880, *T. evansi* has been reported in a broad array of domesticated species – including buffaloes, camels, cattle, and dogs – as well as wildlife such as bats, deer, and rodents across South and Central America, North Africa, the Middle East, the Indian subcontinent, and Southeast Asia [4]. Other *Trypanosoma* species exhibit distinct host affinities; for instance, *T. brucei* is associated with Nagana disease in cattle in the Americas and Africa, whereas *Trypanosoma* *congolense* and *Trypanosoma* *vivax* typically infect small and large ruminants, and *T. equiperdum* is primarily pathogenic to equines [5].

The wide geographical distribution and expansive host range of these parasites are largely attributed to mechanical transmission through bites from various blood-feeding flies, particularly those of the genera *Tabanus* and *Stomoxys* [1, 6]. Emerging evidence has also identified rodents as potential reservoirs for *Trypanosoma lewisi*, a zoonotic trypanosome, which may play a role in the transmission of surra to humans and warrants further epidemiological attention [7, 8]. Clinically, infections range from acute, often fatal, manifestations to chronic conditions characterized by subcutaneous edema, fever, lethargy, weight loss, miscarriage, mucosal hemorrhages, and stiffness of the limbs. Infected animals may also develop anemia, neuropathies, and immunosuppression, frequently culminating in death. Neurological signs have been observed in a variety of affected species, including horses, camels, buffaloes, cattle, deer, and cats [3, 9].

In Indonesia, investigations into trypanosomiasis in domesticated animals, including buffalo, cattle, horses, and dogs have been ongoing since 1988, with reported incidence rates demonstrating substantial regional variability [10–27].

Although trypanosomiasis has long been recognized as a persistent veterinary and public health concern in Indonesia, most available studies have focused on localized outbreaks or species-specific investigations, often lacking methodological consistency and national-scale integration. The heterogeneous nature of diagnostic techniques, geographical coverage, and temporal scopes among published studies has limited the ability to derive a reliable, country-wide estimate of disease burden. Furthermore, previous reports have seldom employed advanced quantitative methods such as meta-analysis to evaluate the cumulative prevalence and epidemiological trends of trypanosomiasis in domesticated animals. There is also limited evidence on how factors such as animal host species, diagnostic modality, and regional ecology influence disease distribution. The absence of comprehensive, pooled data undermines effective policy-making, disease control, and risk assessment strategies in endemic settings such as Indonesia.

This study aimed to systematically synthesize existing epidemiological data on the prevalence of trypanosomiasis in domesticated animals across Indonesia through a rigorous systematic review and meta-analysis. Specifically, the objectives were to: (i) estimate the pooled national prevalence of trypanosomiasis in major domesticated hosts, (ii) assess spatial and temporal variability in reported prevalence across Indonesian provinces from 1988 to 2024, (iii) evaluate the impact of diagnostic methods and host species on prevalence estimates, and (iv) identify knowledge gaps and potential biases in the existing literature to inform future surveillance and control strategies.

## MATERIALS AND METHODS

### Ethical approval

**Figure 1:**
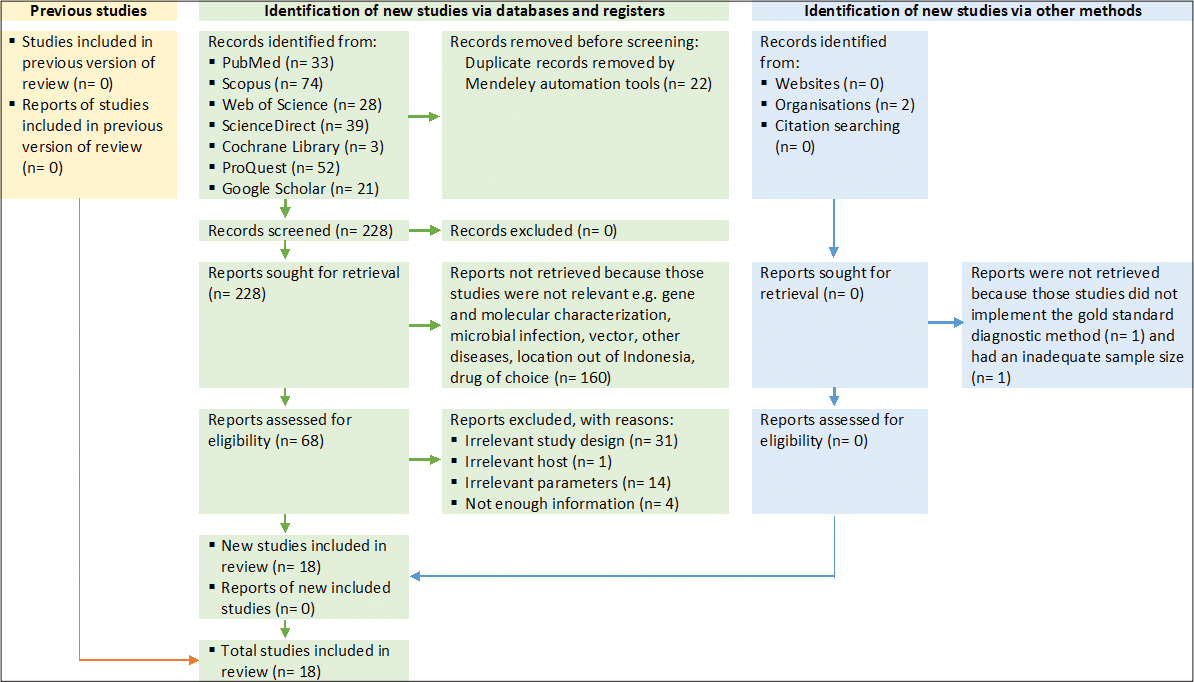
Preferred reporting items for systematic reviews and meta-analyses flow diagram of the study selection process.

The Preferred Reporting Items for Systematic Reviews and Meta-Analyses (PRISMA) 2020 flow diagram was utilized to identify relevant studies for inclusion in this systematic review (Figure 1) [28]. The literature search protocol was registered with the Open Science Framework (OSF). Ethical approval was not required for this study, as it relied exclusively on secondary data from previously published studies and did not involve live animals or laboratory experimental procedures. Consequently, institutional review board approval and informed consent were not applicable.

### Study period and locations

This systematic review and meta-analysis were conducted between October 4 and December 23, 2024. Two research teams based at Universitas Airlangga, Indonesia, and Eskişehir Osmangazi Üniversitesi, Türkiye, performed literature screening, data extraction, statistical analysis, and data visualization independently. Consensus on study findings, data extraction methodologies, and resolution of discrepancies was achieved through scheduled virtual meetings Zoom.

### Search strategy and study selection

A comprehensive literature search was conduct -ed across six major electronic databases – PubMed, Scopus, Web of Science, ScienceDirect, Cochrane Library, and ProQuest to identify relevant studies reporting the prevalence of trypanosomiasis in domesticated animals in Indonesia. The research question was formulated using the PICO framework (P: trypanosomiasis; I: not applicable; C: not applicable; O: prevalence, incidence, or outbreak in Indonesia). The search terms included “Trypanosomiasis,” “prevalence,” “incidence,” and “Indonesia.” Medical Subject Headings (MeSH) and advanced search techniques were employed to optimize search sensitivity and specificity. A sample search algorithm included: #1 “Trypanosomiasis”[MeSH Terms] OR “surra” [Title/Abstract] OR “*Trypanosoma evansi*” [Title/Abstract] OR “*Trypanosoma equiperdum*” [Title/Abstract] OR “*Trypanosoma vivax*” [Title/Abstract] OR “*Trypanosoma brucei*” [Title/Abstract]; AND #2 “outbreak” [Title/Abstract] OR “incidence” [Title/Abstract] OR “prevalence” [Title/Abstract] OR “Indonesia” [Title/Abstract] OR “Java” [Title/Abstract] OR “Sumatra” [Title/Abstract] OR “Kalimantan” [Title/Abstract] OR “Sulawesi” [Title/Abstract] OR “Papua” [Title/Abstract] OR “Bali” [Title/Abstract] OR “Nusa Tenggara” [Title/Abstract].

### Eligibility criteria

Following the removal of duplicate entries using Mendeley software version 1.19.5 (Mendeley Ltd., Elsevier, Netherlands), titles and abstracts were screened against predefined inclusion and exclusion criteria. Studies were considered eligible if they: (i) reported the prevalence of trypanosomiasis in domesticated animals in Indonesia; (ii) provided primary prevalence data; and (iii) employed identifiable diagnostic methods such as enzyme-linked immunosorbent assay (ELISA), polymerase chain reaction (PCR), card agglutination trypanosomiasis test (CATT), or Microhematocrit Centrifuge Technique (MHCT). Exclusion criteria included studies that were (i) reviews, case reports, editorials, or commentaries; (ii) conducted outside Indonesia; (iii) lacking prevalence data; or (iv) focused on non-domesticated animal species. No restrictions were imposed on publication language or study year. The study selection adhered to the PRISMA 2020 flow diagram (Figure 1).

### Data extraction

Data extraction was performed independently by three reviewers (LWF, FF, and MTEP), with disagreements resolved through consultation with a fourth reviewer (HÇ). A fifth researcher (APW) cross-validated the extracted data for consistency and accuracy. Extracted variables included author names, year of publication, study period, study location, province, number of positive events, sample sizes, calculated prevalence rates, host species, and diagnostic methods used. Data discrepancies were carefully evaluated and harmonized among the researchers. All extracted information was systematically tabulated using Microsoft Excel (Microsoft Corp., Redmond, WA, USA). The geospatial distribution of case reports was mapped using QGIS version 3.22.8 (QGIS Association; Białowieża https://qgis.org/).

### Statistical analysis

Statistical analyses were conducted using the “meta” package in R Studio software version 4.4.2 (Posit PBC, USA). Data for “events” and “sample size” were categorized as dichotomous variables, whereas the prevalence rates were treated as continuous variables. Random-effect models were employed, with log odds ratios calculated to synthesize cumulative prevalence estimates. Heterogeneity among studies was quantified using Tau-squared (τ^2^) and I^2^ statistics. An I^2^ value >50% and a p < 0.05 were considered indicative of substantial heterogeneity. Meta-regression was conducted to explore sources of heterogeneity, and results were visualized using scatter plots displaying 95% prediction intervals and 95% confidence intervals (CI). Subgroup analyses were performed based on the study period, geographic location, host species, and diagnostic method. Publication bias was assessed through funnel plots and Egger’s test, utilizing the “metafor” package in R Studio software version 4.4.2 (Posit PBC, USA).

## RESULTS

### Identification of studies

For this systematic review and meta-analysis, a total of 250 studies were initially retrieved from seven electronic databases: PubMed (33 articles), Scopus (74 articles), Web of Science (28 articles), ScienceDirect (39 articles), Cochrane Library (three articles), ProQuest (52 articles), and Google Scholar (21 articles). Following the removal of duplicate records and the initial screening process, 228 publications met the eligibility criteria and were subjected to further review. Among these, 160 articles were excluded primarily because they concerned unrelated topics such as gene and molecular characterization, microbial infection, vectors, other diseases, studies outside of Indonesia, or drug-related research. An additional 50 studies were excluded due to irrelevant study designs (n = 31), inappropriate host species (n = 1), unsuitable parameters (n = 14), and insufficient information (n = 4). The detailed process of study selection is presented in Figure 1.

### Characteristics of the included studies

Ultimately, 18 articles m*et al*l inclusion criteria and were incorporated into the quantitative synthesis for meta-analysis. Collectively, these studies analyzed 4,295 samples, with individual study sample sizes ranging from 27 to 1,080. Samples were distributed across five studies from East Nusa Tenggara (n = 1,080), three studies from South Sulawesi (n = 246), two from Aceh (n = 620), three from Central Java (n = 449), two from East Java (n = 341), and one each from Southeast Sulawesi (n = 27), North Sulawesi (n = 223), South Kalimantan (n = 388), Central Kalimantan (n = 44), Bali (n = 275), West Nusa Tenggara (n = 114), Lampung (n = 302), Jakarta (n = 28), Yogyakarta (n = 101), and West Java (n = 57).

Regarding host species, 11 studies involved cattle (n = 2,629), six studies involved horses (n = 810), six studies involved buffaloes (n = 799), and one study involved dogs (n = 57). Diagnostic techniques employed across the studies included ELISA (n = 2,885 samples), CATT (n = 950), MHCT (n = 259), and PCR (n = 201) (Table 1) [10–27].

**Table 1:** Characteristics of included studies.

Study period	City	Province	Events	Sample size	Prevalence (%)	Host	Test	Reference
2020	Muna	Southeast Sulawesi	15	27	55.56	Cattle	ELISA	[10]
N/A	Denpasar	Bali	0	275	0	Cattle	CATT	[11]
1995	Batang	Central Java	11	30	35	Buffalo	CATT	[12]
1995	Pekalongan	Central Java	26	65	40	Buffalo	CATT	
1995	Pemalang	Central Java	28	50	56	Buffalo	CATT	
1995	Tegal	Central Java	28	56	50	Buffalo	CATT	
1995	Brebes	Central Java	21	38	54	Buffalo	CATT	
N/A	West Lombok	West Nusa Tenggara	16	74	21.6	Cattle	CATT	[13]
N/A	East Lombok	West Nusa Tenggara	7	20	35	Cattle	CATT	
N/A	Central Lombok	West Nusa Tenggara	7	20	35	Cattle	CATT	
2017	Jeneponto	South Sulawesi	2	65	3.07	Horse	MHCT	[14]
N/A	East Sumba	East Nusa Tenggara	8	100	8	Horse	MHCT	[15]
2018	Jakarta	Jakarta	1	28	3.6	Dog	PCR	[16]
2018	Yogyakarta	Yogyakarta	3	29	10.3	Dog	PCR	
2017	West Sumba	East Nusa Tenggara	8	48	16.7	Horse	CATT	[17]
2017	Southwest Sumba	East Nusa Tenggara	5	50	10	Horse	CATT	
2017	Middle Sumba	East Nusa Tenggara	6	43	13.9	Horse	CATT	
2017	East Sumba	East Nusa Tenggara	9	70	12.9	Horse	CATT	
N/A	Pidie	Aceh	6	10	60	Cattle	ELISA	[18]
N/A	Aceh Utara	Aceh	67	153	44	Cattle	ELISA	
N/A	Aceh Besar	Aceh	32	84	38	Cattle	ELISA	
N/A	Aceh Barat	Aceh	8	15	53	Cattle	ELISA	
N/A	Aceh Timur	Aceh	20	45	44	Cattle	ELISA	
N/A	Madura	East Java	39	130	30	Cattle	ELISA	[19]
N/A	Madura	East Java	69	147	47	Buffalo	ELISA	
N/A	N/A	Aceh	134	287	46.7	Cattle	ELISA	[20]
N/A	East Sumba	East Nusa Tenggara	33	184	17.9	Cattle	ELISA	
N/A	Lampung	Lampung	174	271	64.2	Cattle	ELISA	
N/A	N/A	South Sulawesi	16	50	32	Cattle	ELISA	
N/A	N/A	North Sulawesi	30	81	37	Cattle	ELISA	
N/A	Timor	East Nusa Tenggara	111	291	38.1	Cattle	ELISA	
N/A	N/A	South Kalimantan	152	358	42.5	Cattle	ELISA	
N/A	East Sumba	East Nusa Tenggara	32	112	28.6	Buffalo	ELISA	
N/A	Lampung	Lampung	27	31	87.1	Buffalo	ELISA	
N/A	N/A	South Kalimantan	3	30	10	Buffalo	ELISA	
N/A	N/A	Central Java	72	103	70	Buffalo	ELISA	
N/A	Aceh	Aceh	1	26	3.8	Horse	ELISA	
N/A	East Sumba	East Nusa Tenggara	0	41	0	Horse	ELISA	
N/A	N/A	South Sulawesi	1	31	2.8	Horse	ELISA	
N/A	N/A	North Sulawesi	5	142	3.2	Horse	ELISA	
N/A	N/A	Central Java	2	107	1.7	Horse	ELISA	
N/A	N/A	West Java	3	15	20	Cattle	ELISA	[21]
N/A	N/A	West Java	6	11	54.5	Buffalo	ELISA	
1986–1987	Bantul	Yogyakarta	23	30	77	Buffalo	ELISA	[22]
1986–1987	Sleman	Yogyakarta	26	39	67	Buffalo	ELISA	
1986–1987	Kulon Progo	Yogyakarta	2	3	67	Buffalo	ELISA	
1986–1987	Garut	West Java	21	31	68	Buffalo	ELISA	
N/A	N/A	Central Kalimantan	33	44	75	Cattle	PCR	[23]
2019	East Sumba	East Nusa Tenggara	0	57	0	Horse	CATT	[24]
2019	East Sumba	East Nusa Tenggara	5	23	21.7	Buffalo	CATT	
2019	East Sumba	East Nusa Tenggara	3	31	9.7	Cattle	CATT	
2019–2020	Makassar	South Sulawesi	3	100	3	Cattle	PCR	[25]
2018	Banyuwangi	East Java	0	64	0	Cattle	MHCT	[26]
2023	East Sumba	East Nusa Tenggara	6	30	20	Horse	MHCT	[27]

N/A=Data not available, ELISA=Enzyme-linked immunosorbent assay, CATT=Card agglutination trypanosomiasis test, PCR=Polymerase chain reaction, MHCT=Microhematocrit centrifuge technique

**Figure 2:**
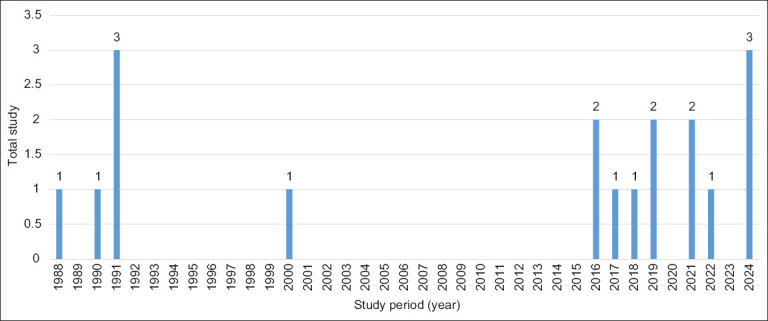
Distribution of studies relevant to the prevalence of trypanosomiasis in domesticated animals in Indonesia since 1988.

**Figure 3:**
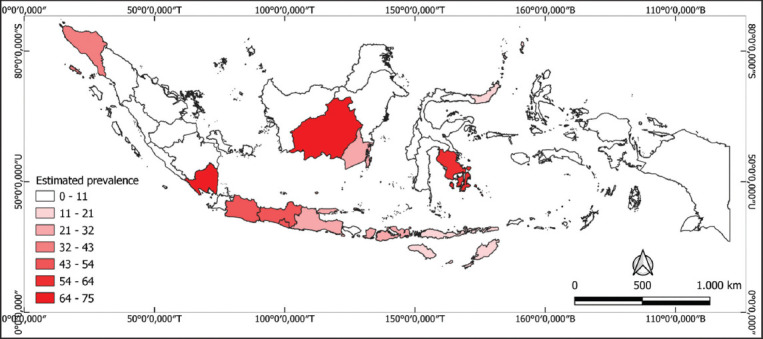
The distribution of risk areas for trypanosomiasis in domesticated animals in Indonesia based on studies conducted since 1988. The distribution of study reports was visualized using QGIS v3.22.8.

The first report of trypanosomiasis in cattle in Indonesia was published in Aceh in 1988. Between 2016 and 2022, fluctuations in prevalence were consistently reported, with most surveillance studies published between 1991 and 2024 (Figure 2). Geospatial data visualizations depicting the distribution of trypanosomiasis prevalence across Indonesian provinces were developed, with the highest prevalence estimates (66.7%–75%) depicted in red and the lowest estimates (0%–8.3%) or unreported regions shown in white (Figure 3).

### Overall pooled prevalence

**Figure 4:**
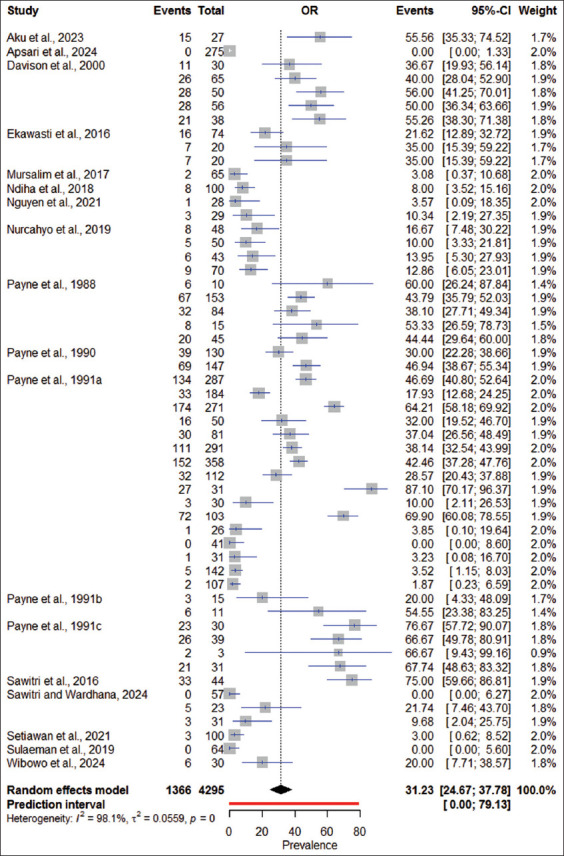
Forest plot of pooled prevalence and 95% confidence interval of trypanosomiasis in domesticated animals in Indonesia across studies.

Across all 18 studies, the overall pooled prevalence of trypanosomiasis in domesticated animals in Indonesia was estimated at 31.23% (95% CI: 24.67%–37.78%). A high level of heterogeneity was observed among the included studies (I^2^ = 98.1%, τ^2^ = 0.0559, p = 0). The meta-analysis demonstrated that the incidence of trypanosomiasis varied across the provinces that implemented surveillance between 1988 and 2024, with a predicted prevalence range of 0.00%–79.13%. Notably, between 2018 and 2024, a reduced fluctuation trend was observed, with prevalence ranging from 4.29% to 28.44% (Figure 4).

### Subgroup analysis

Subgroup meta-analyses identified significant differences across four key variables: study period (p < 0.0001), study location (p < 0.0001), animal host (p < 0.0001), and diagnostic method (p < 0.0001).

The highest pooled prevalence by study period was recorded in 1987, at 70.34% (95% CI: 61.46%–79.11%). Over the past two decades, the highest incidence was reported in 2016, at 41.86% (95% CI: 18.00%–66.71%). Lampung and Central Kalimantan provinces exhibited the highest prevalence estimates, with 75.05% (95% CI: 52.64%–97.45%) and 75.00% (95% CI: 59.66%–86.81%), respectively.

Other provinces with higher than average prevalence included Southeast Sulawesi (55.56%), Yogyakarta (53.47%), West Java (47.46%), Central Java (43.87%), and Aceh (39.50%).

Regarding host species, buffaloes had the highest prevalence estimate at 51.46% (95% CI: 40.71%–62.20%), followed by cattle at 33.99% (95% CI: 25.48%–42.50%). In terms of diagnostic techniques, ELISA demonstrated the highest sensitivity, with a pooled prevalence of 39.10% (95% CI: 30.13%–48.07%), followed by CATT with 24.86% (95% CI: 15.62%–34.10%) (Table 2).

**Table 2:** The overall pooled prevalence of trypanosomiasis in domesticated animals in Indonesia and subgroup meta-analysis.

Categories	Total studies or subgroups	Prevalence (%)		Heterogeneity			p-value for subgroup difference
	
		Estimate	95% CI	I^2^ (%)	τ^2^	p-value	
Overall	18	31.23	24.67–37.78	98.1	0.0559	0	
Study period							
• 1987	1	70.34	61.46–79.11	0	0	0.7959	<0.0001
• 1988	1	43.27	37.75–48.79	0	0	0.5966	
• 1990	1	38.45	21.85–55.05	88.5	0.0127	0.0032	
• 1991	2	30.76	18.63–42.88	98.5	0.0660	<0.0001	
• 1995	1	47.64	40.24–55.04	28.2	0.0019	0.2335	
• 2016	2	41.86	18.00–66.71	93.2	0.0522	<0.0001	
• 2017	2	10.17	4.76–15.57	62.9	0.0021	0.0290	
• 2018	3	4.29	0.00–9.08	71.3	0.0015	0.0151	
• 2019	1	8.23	0.00–19.95	78.1	0.0080	0.0103	
• 2020	2	28.44	0.00–79.91	96.6	0.1334	<0.0001	
• 2023	1	20.00	7.71–38.57	N/A	N/A	N/A	
• 2024	1	0	0.00–1.33	N/A	N/A	N/A	
Province							
• Lampung	1	75.05	52.64–97.45	91.5	0.0240	0.0006	<0.0001
• Central Kalimantan	1	75.00	59.66–86.81	N/A	N/A	N/A	
• Southeast Sulawesi	1	55.56	35.33–74.52	N/A	N/A	N/A	
• Yogyakarta	2	53.47	20.62–86.32	95.2	0.0960	<0.0001	
• West Java	2	47.46	18.16–76.76	84.6	0.0542	0.0015	
• Central Java	2	43.87	26.88–60.86	98.3	0.0485	<0.0001	
• Aceh	2	39.50	25.62–53.38	93.7	0.0292	<0.0001	
• West Nusa Tenggara	1	27.01	16.71–37.31	10.9	0.0023	0.3257	
• South Kalimantan	1	26.59	0.00–58.39	96.5	0.0508	<0.0001	
• East Java	2	25.41	0.00–52.48	98.8	0.0561	<0.0001	
• North Sulawesi	1	19.89	0.00–58.39	96.5	0.0508	<0.0001	
• East Nusa Tenggara	5	14.78	8.50–21.06	94.7	0.0113	<0.0001	
• South Sulawesi	3	9.23	0.00–21.87	83.9	0.0153	0.0003	
• Jakarta	1	3.57	0.09–18.35	N/A	N/A	N/A	
• Bali	1	0	0.00–1.33	N/A	N/A	N/A	
Host							
• Cattle	11	33.99	25.48–42.50	98.7	0.0390	0	<0.0001
• Buffalo	6	51.46	40.71–62.20	91.4	0.0408	<0.0001	
• Horse	6	5.46	2.62–8.31	68.8	0.0017	0.0001	
• Dog	1	5.51	0.00–11.50	3.5	<0.0001	0.3087	
Test							
• ELISA	6	39.10	30.13–48.07	98.0	0.0574	<0.0001	<0.0001
• CATT	5	24.86	15.62–34.10	95.1	0.0314	<0.0001	
• PCR	3	22.66	0.00–56.46	97.4	0.1167	<0.0001	
• MHCT	4	5.38	0.00–11.43	79.4	0.0028	0.0023	

95% CI=95% confidence intervals, I^2^=The primary index for reporting heterogeneity, τ^2^=Tau-squared focuses on the variability of true effect sizes, N/A=Data not available, ELISA=Enzyme-linked immunosorbent assay, CATT=Card agglutination trypanosomiasis test, PCR=Polymerase chain reaction, MHCT=Microhematocrit centrifuge technique

### Meta-regression and publication bias

**Figure 5:**
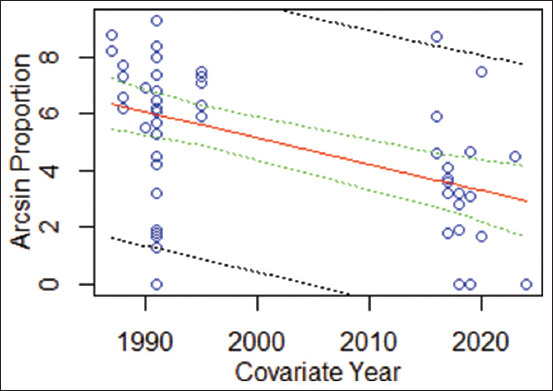
Scatter plot of the meta-regression analysis to evaluate trends in the prevalence of trypanosomiasis in domesticated animals in Indonesia since 1988. The red line (---) represents the regression line, the green line (---) represents the 95% confidence interval, and the black line (---) represents the 95% prediction interval.

Meta-regression analysis revealed a statistically significant association between study year and trypanosomiasis prevalence in domesticated animals in Indonesia (−0.09208x + 189.29336 * Year; 95% CI: −5.9719–5.0300; R^2^ = 0.2178; p = 0.0002221) (Figure 5). Cumulative meta-analysis illustrated temporal varia- tions in the pooled prevalence estimates. Following an initial peak prevalence of 70.34% (95% CI: 61.46%–79.11%) in 1987, prevalence declined to 30.76% (95% CI: 18.63%–42.88%) in 1991, then rose again to 47.64% (95% CI: 40.24%–55.04%) in 1995. A general decreasing trend was noted between 2016 and 2024, with an estimated prevalence ranging between 0% and 41.86%.

**Figure 6:**
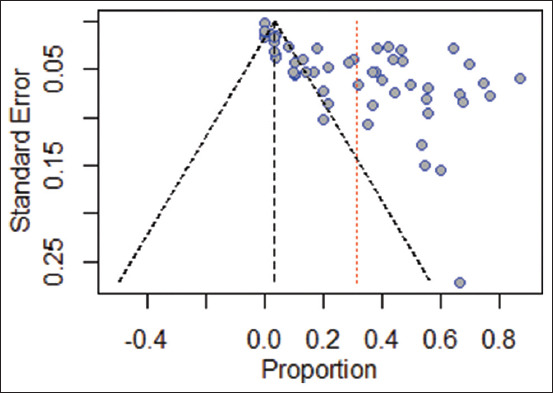
Funnel plot of standard error for evaluating publication bias in the prevalence of trypanosomiasis in domesticated animals in Indonesia across studies.

Evaluation of publication bias through funnel plot visualization (Figure 6) revealed no apparent asymmetry. However, Egger’s test indicated potential publication bias in the included studies (p < 0.0001).

## DISCUSSION

### High heterogeneity and temporal fluctuations

The findings of this meta-analysis revealed a substantial degree of heterogeneity among the included studies (I^2^ = 98.1%, τ^2^ = 0.0559, p = 0). Since 1988, the reported prevalence of trypanosomiasis in domesticated animals across Indonesia has exhibited significant tem-poral fluctuations, ranging from 0.00% to 79.13%. Among the host species evaluated, buffaloes demon-strated the highest pooled prevalence at 51.46% (95% CI: 40.71%–62.20%), followed by cattle, horses, and dogs.

The elevated prevalence among buffaloes is likely influenced by their widespread distribution and extensive use in agricultural activities, particularly in regions where they constitute up to 70%–95% of livestock populations [12, 19–22, 24]. Most rural communities retain mature female buffaloes for draught power, and these animals are frequently exposed to fly vectors during grazing or when working in rice fields for prolonged periods (1–6 months annually). Environmental exposure and large herd sizes – sometimes exceeding 100 animals – may facilitate the transmission of *T. evansi*.

### Host-specific risk profiles

Differences in host species distribution may influence local infection dynamics. In provinces where cattle, horses, and buffaloes were sampled simultaneously, buffaloes consistently demonstrated higher infection rates. This may be attributed to vector ecology, such as the abundance of tabanid flies in irrigated rice field areas or the possible host preference of these vectors [19–21, 24].

In contrast, horses exhibited lower infection rates, potentially due to reduced exposure to infected hosts and environmental vectors in urban settings. In addition, horses’ behavioral responses, such as swatting and mobility may deter fly feeding. Nevertheless, horses used for labor in endemic areas may require preventive chemoprophylaxis or therapeutic interventions [17, 20].

### Geographic variation in prevalence

Subgroup analyses based on study location identified considerable geographic variability. Lampung (75.05%, [95% CI: 52.64%–97.45%]) and Central Kalimantan (75.00%, [95% CI: 59.66%–86.81%]) exhibited the highest regional prevalence estimates. In contrast, trypanosomiasis was infrequently detected in South Sulawesi (9.23%, [95% CI: 0.00%–21.87%]), Jakarta (3.57%, [95% CI: 0.09%–18.35%]), and Bali (0%, [95% CI: 0.00%–1.33%]).

Vector ecology and environmental factors may explain this disparity. Areas with high annual rainfall – such as Lampung (1,883 mm) – are known to support larger tabanid populations, increasing the risk of transmission. Conversely, arid zones such as East Sumba (768 mm annual rainfall) report lower infection rates [20]. Sanitation practices, livestock density, and the effectiveness of vector control measures also vary significantly across provinces, influencing disease dynamics [29].

### Climatic and management-related risk factors

Environmental parameters such as air temperature, relative humidity, and wind speed directly influence fly activity. Tabanid flies are most active at moderate humidity (~35%) and high temperatures (~32°C), whereas low activity is recorded under high humidity (~80%) or cooler temperatures (~18°C). Wind speeds above 10 km/h have been shown to significant- ly reduce vector abundance [30].

Migration, elevation, and land-use practices also modulate trypanosomiasis epidemiology. Effective disease control strategies must consider regional differences in livestock management systems, fly vector ecology, and the role of environmental stressors [31].

### Risk awareness and treatment practices

In Sumba, Indonesia, a previous study by Nurcahyo *et al*. [17] identified livestock origin, sex, species, management practices, and farmers’ knowledge as critical risk factors for trypanosomiasis transmission. Communal grazing was associated with higher infec- tion rates [32]. The prevalent use of single trypanoci-dal drugs without integrated vector management may contribute to the emergence of drug resistance and increased disease burden. Studies suggest that combining chemotherapeutic agents with vector control strategies results in a significant reduction in infection rates. Treating cattle with insecticides can improve cost-benefit outcomes by targeting both the parasite and its vector [33].

### Diagnostic techniques and their limitations

Four diagnostic techniques – ELISA, CATT, PCR, and MHCT – were employed across the studies, each with distinct strengths and limitations. ELISA was the most frequently applied method (39.10%, [95% CI: 30.13%–48.07%]) and effectively detected antibodies against *T. evansi* across multiple provinces [10, 18, 20, 22]. While sensitive, ELISA primarily detects past or ongoing infections and cannot differentiate between species.

PCR-based diagnostics demonstrated a lower pooled prevalence (22.66%, [95% CI: 0.00%–56.46%]) but offered high specificity. The RoTat 1.2 versus gene and Internal Transcribed Spacer (ITS)-1/ITS-2 regions were targeted for detection in dogs and cattle in Jakarta, Yogyakarta, and South Sulawesi [16, 23, 25]. These methods provide accurate identification of *T. evansi* strains and are less prone to cross-reactivity, making them ideal for molecular surveillance [34, 35].

CATT was widely used due to its cost-effectiveness and operational simplicity (24.86%, [95% CI: 15.62%–34.10%]). However, its utility is limited by variations in immunoglobulin M levels, which may lead to false negatives or positives depending on the infection stage and immune complex formation. Furthermore, cross-reactivity with other *Trypanozoon* species has been reported, limiting its specificity in cattle and pigs [36–38].

The MHCT method showed the lowest prevalence estimate (5.38%, [95% CI: 0.00%–11.43%]) and reflects the presence of patent parasitemia. Given the fluctuating parasitemia in chronic infections, MHCT may underreport true infection rates. Nevertheless, it is useful for identifying animals with high blood trypanosome levels who are more likely to serve as reservoirs for vector-borne transmission [20].

## CONCLUSION

This systematic review and meta-analysis comprehensively assessed the prevalence of trypanosomiasis in domesticated animals across Indonesia between 1988 and 2024. The pooled prevalence was estimated at 31.23% (95% CI: 24.67%–37.78%), with substantial heterogeneity observed across provinces, host species, diagnostic methods, and study periods. Buffaloes exhibited the highest infection rates (51.46%), and Lampung and Central Kalimantan recorded the greatest regional prevalence (>75%). Although the overall prevalence demonstrated a decreasing trend over the past two decades, localized surges persist, highlighting the disease’s continued epidemiological significance.

The findings underscore the urgent need for enhanced surveillance, targeted vector control measures, and the integration of standardized, highly sensitive diagnostic techniques such as PCR and ELISA in endemic regions. These interventions are critical not only for reducing animal morbidity and economic losses in the livestock sector but also for mitigating potential zoonotic risks associated with *T. evansi* transmission.

A major strength of this study lies in its national coverage, incorporating diverse temporal and geographical datasets using rigorous meta-analytic methods. However, limitations include potential publication bias, uneven regional data availability, and the exclusion of gray literature and unpublished studies, which may have influenced the estimated prevalence rates. Furthermore, diagnostic variability among studies could have introduced inconsistencies in case detection.

Future research should prioritize longitudinal studies employing molecular diagnostics to monitor infection dynamics more accurately. Investigations into environmental, ecological, and anthropogenic risk factors influencing vector distribution are warranted. In addition, developing integrated disease management strategies combining chemotherapeutic interventions, vector control, and farmer education programs will be essential to sustainably reduce the burden of trypanosomiasis in Indonesia. A One Health approach, recognizing the interconnectedness of animal, human, and environmental health, should guide future surveillance and control initiatives.

## AUTHORS’ CONTRIBUTIONS

MTEP: Designed and developed the research methodology. After screening the eligible study, LWF, FF, APW, HÇ, and MTEP: Collected, curated, and extracted the data. FF, HÇ, and MTEP: Contributed to the data analysis, validation, and tables and figures, and QGIS map visualization. LWF, HÇ, and MTEP: Wrote the draft, revised, and submitted the manuscript. All authors read and approved the final manuscript.
